# Health Monitoring of Fattening Pigs – Use of Production Data, Farm Characteristics and On-Farm Examination

**DOI:** 10.1186/s40813-021-00225-y

**Published:** 2021-08-03

**Authors:** Julia Grosse-Kleimann, Birte Wegner, Ines Spiekermeier, Elisabeth grosse Beilage, Nicole Kemper, Hendrik Nienhoff, Heiko Plate, Henning Meyer, Hubert Gerhardy, Lothar Kreienbrock

**Affiliations:** 1 Department of Biometry, Epidemiology and Information Processing, WHO Collaborating Centre for Research and Training for Health in the Human-Animal-Environment Interface, University of Veterinary Medicine Hannover, Foundation, Hanover, Germany; 2 Institute for Animal Hygiene, Animal Welfare and Farm Animal Behaviour, University of Veterinary Medicine Hannover, Foundation, Hanover, Germany; 3 Swine Health Service, Chamber of Agriculture in Lower Saxony, Oldenburg, Germany; 4 Field Station for Epidemiology, University of Veterinary Medicine Hannover, Foundation, Bakum, Germany; 5 VzF e.V, Association for Promoting Farming Economics, Uelzen, Germany; 6 MSG, Marketing Service Gerhardy, Garbsen, Germany

**Keywords:** farm management, secondary data use, welfare indicators, swine

## Abstract

**Background:**

The use of processed secondary data for health monitoring of fattening pigs has been established in various areas, such as the use of antibiotics or in the context of meat inspection. Standardized scores were calculated based on several sources of production data and can be used to describe animal health in a large collective of pig units. In the present study, the extent to which these scores are related to different farm characteristics and management decisions were investigated. In addition, slaughter scores were compared with the results of a veterinary examination on the farms.

**Results:**

The comparison of the results of the uni- and multifactorial analyses revealed that almost all of the examined factors play a role in at least one of the scores when considered individually. However, when various significant influencing factors were taken into account at any one time, most of the variables lost their statistical significance due to confounding effects. In particular, production data such as production costs or daily feed intake remained in the final models of the scores on mortality, average daily gain and external lesions. Regarding the second part of the investigation, a basic technical correlation between the slaughter scores and the on-farm indicators could be established via principal component analysis. The modelling of the slaughter scores by the on-farm indicators showed that the score on external lesions could be represented by equivalent variables recorded on the farm (e.g., lesions caused by tail or ear biting).

**Conclusions:**

It has been demonstrated that the examined health scores are influenced by various farm and management characteristics. However, when several factors are taken into account, confounding occurs in some cases, which must be considered by consultants. Additionally, it was shown that on-farm examination content is related to the scores based on equivalent findings from slaughter pigs.

**Supplementary Information:**

The online version contains supplementary material available at 10.1186/s40813-021-00225-y.

## Background

Measuring animal welfare and animal health has steadily increased in importance recently. Many methods have been developed, and a rough distinction, following the EFSA [1], can be made between animal-based, active indicators and passive, environment-based indicators. The latter can be used if their relation to animal health issues is very close and their recording is easier, which is important for monitoring the health status of a wide range of pig farms. GrosseKleimann et al. [2] have already derived a set of seven health scores and one total score for this purpose, which allows longitudinal screening of a target collective of farms under conventional production conditions in Germany. One-half of the scores is based on findings in slaughtered pigs, whereas the other half consists of information regarding biological performance (average daily gain and feed conversion ratio), mortality and treatment with antibiotics. The data were generated from monitoring and advisory processes along the pig production chain, and both were checked and processed for secondary data use beforehand.

Concerning the aforementioned health scores [2], the question is whether management and farm characteristics could influence them and whether particular factors for the prevention of health incidents can be identified. Several studies have addressed this issue and found production items that can be used as a basis for extended veterinary and agricultural consulting to improve the health status of fattening pigs in the long run. However, these factors may vary by production system or the particular collective of farms. Therefore, this study will describe which farm characteristics can be used as adjusting screws for the health scores in fattening pigs housed in typical German pig production systems .

Additionally, this investigation addresses the degree to which health scores, which are based on findings in slaughtered pigs, can be reflected or complemented by animal welfare indicators that are collected on farms in fattening pigs.

## Methods

### Study design

The joint project “Multivariate Assessment of Animal Welfare through Integrative Data Collection and Validation of Welfare Indicators in Finishing Pigs” (MulTiViS) is conducted by a consortium of the University of Veterinary Medicine Hannover, foundation (“Stiftung Tierärztliche Hochschule Hannover”), the Swine Health Service of the Chamber of Agriculture in Lower Saxony (“Schweinegesundheitsdienst”), the swine service provider VzF (registered association, VzF) and Marketing Service Gerhardy. The owners of a sample of 207 commercial fattening pig units from northeastern Lower Saxony, Germany, all under the advisory service of VzF, were asked for their written confirmation of participation.

For each pig unit, several sources of production data were available, acquired by VzF in the context of farm branch analysis. These production data contain information on various farm and management characteristics, biological and economic performance data, findings in slaughtered pigs and information on antibiotic usage.

Additionally, two trained veterinarians visited each farm on one day between 3 and 2017 and 12 November 2018. They examined fattening pigs in a maximum of eight randomly selected pens per farm (7.8 ± 1; arithmetic mean ± standard deviation), according to the method of Kish, 1949 [3], which led to 24,715 observed animals in 1,201 pens. Thirty welfare and health indicators were investigated as on-farm health information. To achieve a holistic “animal health dataset”, on-farm health information was linked to production data from the corresponding half-year in which the on-farm examination took place.

Since Grosse-Kleimann et al. [2] found the initial body weight (IBW) on day one of the fattening period to be an important stratification criterion, only herds with IBW between 24 and 33.5 kg were chosen for further analyses. Additionally, data from pigs housed in pens with straw bedding or outdoor climate were excluded because the number of pens was too small to be representative (4 farms with exclusively straw bedding or outdoor climate). Furthermore, on one farm, on the day of the examination, unexpectedly, no animals were present. Finally, a collective of n = 154 pig units remained for further analyses. The farm size varied between 160 and 3,360 (1,153 ± 582) fattening pig places (FPs).

### Health scores

Seven standardized health scores and one total score according to Grosse-Kleimann et al. [2] were chosen as the target variables: mortality (MOR), average daily gain (ADG), feed conversion ratio (FCR), treatment frequency (TF) and meat inspection indicators associated with respiratory health (RESP), exterior injuries or alterations (EXT) and animal management (MANG). They are based on selected indicators from production data (Table 1) and were standardized on a zscale, with a mean of 0 and a standard deviation of 1. For respiratory slaughter findings of pneumonia and pleurisy, in Germany, four categories are usually documented at meat inspection. For the study, a unique prevalence was calculated by combining moderate and high alterations as positive records and slight and no alterations as negative records.

**Table 1:** Source and descriptive measures of indicators, which are the basis for examined health scores in the study collective of 154 pig units, according to Grosse-Kleimann et al. [2]. (*MOR* mortality score, *ADG* average daily gain score, *FCR* feed conversion ratio score, *TF* treatment frequency score, *RESP* respiratory lesions score, *EXT* exterior lesions score, *MANG* animal management score, *UDD* used daily doses, *FP* fattening pig place)

score	Indicator	source	mean	SD	min	max
**MOR**	mortality [%]	performance data	2.56	1.77	0.17	13.73
**ADG**	average daily gain [g]	performance data	849	77	631	1,036
**FCR**	feed conversion ratio [kg/kg]	performance data	2.83	0.19	2.45	3.34
**TF**	treatment frequency [UDD/FP]	antibiotic usage data	2.22	3.57	0.00	20.13
**RESP**	pneumonia [%]	findings in slaughtered pigs	11.99	8.52	0.65	80.82
	pleurisy [%]	findings in slaughtered pigs	6.43	6.18	0.00	29.92
	pericarditis [%]	findings in slaughtered pigs	3.69	3.20	0.00	21.83
**EXT**	arthritis [%]	findings in slaughtered pigs	0.58	0.48	0.00	2.43
	abscess [%]	findings in slaughtered pigs	0.97	0.68	0.00	3.67
	ear lesions [%]	findings in slaughtered pigs	0.01	0.05	0.00	0.37
	tail lesions [%]	findings in slaughtered pigs	0.76	1.12	0.00	7.42
	dermal alterations [%]	findings in slaughtered pigs	0.16	0.23	0.00	1.54
	bursitis [%]	findings in slaughtered pigs	0.46	0.70	0.00	6.14
**MANG**	liver milk spots [%]	findings in slaughtered pigs	6.89	10.69	0.00	61.16
	dermal damage (handling) [%]	findings in slaughtered pigs	0.05	0.24	0.00	2.30
	intestinal alteration [%]	findings in slaughtered pigs	0.60	1.18	0.00	13.93
	whole carcass condemnation [%]	findings in slaughtered pigs	0.17	0.21	0.00	1.05

### Factors selected from farm characteristics

Sixteen categorical factors, e.g., type of buying in criteria, boar fattening or feeding techniques, and 14 metric factors, e.g., costs for veterinary service or daily feed intake, were extracted from farm characteristics (for a complete list of all factors see Additional file 1). Factor levels with less than ten pig units were omitted for statistical analyses to avoid extended variation due to sparse data.

To investigate both the individual influence of farm characteristics on the health scores and the interaction with others, uni- and multifactorial ANOVA and linear regression models were conducted. To find the set of variables in FC that showed the most significant influence on each health score, multifactorial analyses of covariance were conducted with *a combination of forward selection (entering criterion: p-value < 0.1) and manual* backward selection (removal criterion: pvalue > 0.05).

In preparation for statistical modelling and to control multicollinearity, association analyses within influencing factors were previously applied to all variables [4].

### Preparation and analysis of on-farm indicators

During investigation on the farms, two different age groups of pigs were distinguished: first to sixth fattening weeks (age group 1) and seventh to twelfth fattening weeks (age group 2). Since regrouping of pigs for fattening and mixing of unfamiliar pigs often cause rank fights [5, 6], an increased incidence of health- and production-relevant findings may occur in age group 1. In addition, the time until slaughter is longer in age group 1, and thus, the probability is higher that injuries developed in the early fattening period have healed or are no longer visible. Hence, t-tests were carried out to identify those indicators whose mean prevalence differed significantly between the age groups. If differences were identified, for these characteristics, a correction factor for age group 1 was calculated by the following formula:

$${p}_{corr}=p\times \frac{{mean}_{group2}}{{mean}_{group1}}$$

where ${p}_{corr}$ = corrected prevalence of age group 1 and $p$ = observed prevalence of age group 1. This approach is in line with a general adjustment by ratio extrapolation [7].

On-farm health information was originally examined as pen-specific prevalence (number of affected animals per pen), but health scores were calculated at the farm level. To bring them to a uniform level, an average farm-specific prevalence for each on-farm indicator and farm was calculated. To meet the requirements for modelling, logit transformation and z-standardization were applied on all variables following the approach of Nienhaus et al. [8].

To investigate whether the slaughter scores could sufficiently depict pig health or whether investigation of living animals is necessary, several analyses were carried out. A principal component analysis was implemented to provide an overview of the interconnections between on-farm indicators and RESP, EXT and MANG. As the first and second components of the principal component analysis are considered the most discriminating, they were plotted on a factor map. Unifactorial ANOVA for each selected on-farm indicator and three slaughter scores were conducted to examine the degree of predictability and to reveal possible supplemental factors for health monitoring. Previously, the Spearman correlation coefficient was calculated for each on-farm and slaughter indicator.

All statistical evaluations were performed with SAS®, version 9.4 TS level 1M5 (SAS Institute Inc., Cary, NC, United States).

## Results

### Health status (scores)

On average, the mortality was 2.56 % ± 1.77 %, and the mean average daily gain was 849 g ± 77 g. The food conversion ratio ranged between 2.45 kg/kg and 3.34 kg/kg, and the mean number of used daily doses per fattening place was 2.22 ± 3.57. Pneumonia, pleurisy, pericarditis and liver milk spots showed a mean prevalence of 11.99 % ± 8.52 %, 6.43 % ± 6.18 %, 3.69 % ± 3.20 and 6.89 % ± 10.69 %, respectively, whereas all other slaughter indicators occurred only at a very low level (mean prevalence < 1 %). Detailed results are shown in Table 1.

### Farm characteristics and their impact on health scores

Most of the pig units (48.1 %) purchased surgically castrated male pigs that were housed in the same pen as females. The majority of the study farms (46.7 %) offered 0.75 m^2^ per fattening pig place, and 81.8 % had pens that were equipped with fully slatted floors. Regarding feeding techniques, the main type was fully automatic liquid feeding (38.0 %). On average, the production costs varied between 0.57 € and 0.91 € per kg of live weight gain. The mean costs for veterinary service per sold animal were 1.00 € ± 1.17 €, and the feed intake per pig and day was 2.40 kg ± 0.21 kg. Detailed descriptive results are shown in Additional file 1.

In preparation for modelling, the two variables number of FP and live weight at slaughter were excluded for further analyses to avoid multicollinearity. The results of unifactorial ANOVA revealed that three farm characteristics had no significant influence on any of the observed health scores (feed, phase feeding and feed availability). The factor with the highest number of pvalues for entering final models was feed intake per pig and day. Manual backwards selection was performed for the multifactorial models with a p-value < 0.05 as the selection criterion. It was shown that ADG was the most influential health score, with R^2^ = 96.1 % by observed farm characteristics. The results of uni- and multifactorial analyses for MOR, ADG and EXT are shown in Table 2. Detailed results for all scores are listed in Additional file 2.

**Table 2:** Results of unifactorial analyses (factors with pvalue < 0.1) and final multifactorial model (factors with pvalue < 0.05) for MOR (mortality score). R² of final model = 17.54 %. Factors remaining in the final model are in bold letters, and the reference level is in italic letters (*BFCD* benefits free of direct costs, *LWG* live weight gain)

Factor	factor levels	unifactorial		multifactorial	
		**p-value**	**estimate**	**p-value**	**estimate**
**BFDC/100 kg LWG**		< 0.0001	-0.04	< 0.0001	-0.05
**feed energy/kg LWG**		0.0161	0.08	0.0314	0.08
feed availability		0.0570			
	misc.	0.7548	0.09		
	ad libitum	0.0175	0.42		
	*rationed*	*	*		
**costs for veterinary service/pig**		0.0672	0.13	0.0482	0.14
**production costs/kg LWG**		0.0813	2.23	0.0043	-5.59
feed energy		0.0992	0.08		
	< 13 MJ	0.0992	0.28		
	*13–13,4 MJ*	*	*		

* No p-value and estimate for reference level

The results of multifactorial analyses usually revealed no substantial differences in parameter estimates for categorical characteristics in comparison to unifactorial analyses (see Tables 2, 3 and 4). The type of buying in criteria and boar fattening were the variables with the most influence, as they are the only categorical characteristics remaining in a final model. The type of buying in criteria is a variable that contains information on where farms source their piglets from and the management type behind it. The reference group, type A, stands for the typical VzF farm with pigs originating from a German farm and having BHZP (German Federal Hybrid Breeding Programme) as the dominant sow and boar breed. Types B and C also obtain German piglets with BHZP as the main boar breed, but DAN (DanBred P/S) and PIC (Pig Improvement Company) are the primary sow breeds. Types D and E have mixed sow breeds and BHZP or mixed breeds for boar, respectively. Type F obtains imported piglets from Denmark, whereby the sow and boar breed are not taken into account. Type G was created by merging subtypes that were too few for a separate group. Farms with type E buying in criteria seemed to have fewer exterior lesions, whereas type B farms showed better EXT scores than the reference group. In farms with boar fattening, animals showed higher values in EXT than in farms that purchase castrated males.

**Table 3:** Results of unifactorial analyses (factors with pvalue < 0.1) and final multifactorial model (factors with pvalue < 0.05) for ADG (average daily gain score). R² of final model = 96.10 %. Factors remaining in the final model are in bold letters, and the reference level is in italic letters (*BFCD* benefits free of direct costs, *LWG* live weight gain, *FP* fattening pig place)

Factor	factor levels	unifactorial		multifactorial	
		**p-value**	**estimate**	**p-value**	**estimate**
**needed feed/pig/day**		< 0.0001	-3.49	< 0.0001	-4.31
**feed energy/kg LWG**		< 0.0001	0.18	< 0.0001	0.24
group size		< 0.0001			
	misc.	0.0227	0.46		
	21–50 pigs	0.0144	-0.58		
	13–20 pigs	0.0034	-0.56		
	*1–12 pigs*	*	*		
space per FP		< 0.0001			
	> 0.9 m^2^	< 0.0001	-1.14		
	0.825 m²	0.0277	-0.37		
	*0.75 m*^*2*^	*	*		
type of buying in criteria**		0.0001			
	G	0.0142	0.75		
	F	0.0010	-0.99		
	E	0.0507	-0.50		
	D	0.2479	0.30		
	C	0.8341	-0.06		
	B	0.5755	-0.12		
	* A*	*	*		
pigs/FP		0.0011	-0.83		
feeding techniques		0.0022			
	misc.	0.1604	0.38		
	mash	0.0073	-0.46		
	*liquid*	*	*		
water supply		0.0049			
	public	0.0012	-0.56		
	private well, water treated	0.6106	-0.16		
	*private well*	*	*		
**production costs/kg LWG**		0.0083	3.27	< 0.0001	1.29
floor type		0.0145			
	misc.	0.0048	0.70		
	partially slatted	0.3237	0.32		
	*fully slatted*	*	*		
feed availability		0.0192			
	misc.	0.3581	0.26		
	ad libitum	0.0177	-0.40		
	*rationed*	*	*		
live weight of losses		0.0227	-0.02		
boar fattening/single sex		0.0245			
	yes/yes	0.8974	0.03		
	no/yes	0.0103	-0.44		
	*no/no*	*	*		
slaughter weight		0.0272	-0.08		
BFDC/100 kg LWG		0.0311	-0.02		
post fattening		0.0478			
	yes	0.0478	0.37		
	*no*	*	*		
weight gain/pig		0.0890	-0.04		

* No p-value and estimate for reference level

** For explanation of factor levels see “Discussion/Impact of farm and management characteristics”

**Table 4:** Results of unifactorial analyses (factors with pvalue < 0.1) and final multifactorial model (factors with pvalue < 0.05) for **EXT** (exterior lesions score). R² of final model = 26.12 %. Factors remaining in the final model are in bold letters, and the reference level is in italic letters

factor	factor levels	unifactorial		multifactorial	
		**p-value**	**estimate**	**p-value**	**estimate**
type of buying in criteria**		0.0014		0.0024	
	G	0.6629	0.08	0.8411	0.04
	F	0.0011	-0.57	0.0107	-0.47
	E	0.9358	0.01	0.6050	0.08
	D	0.4364	0.12	0.8524	0.03
	C	0.0053	-0.50	0.0010	-0.60
	B	0.5943	-0.07	0.2323	-0.15
	* A*	*	*	*	*
**feed intake/pig/day**		0.0040	-0.64	0.0400	-0.48
phosphor reduction		0.0135			
	yes	0.0135	-0.26		
	*no*	*	*		
costs for disinfection***/pig		0.0137	-0.49	0.0069	-0.53
**boar fattening/single sex**		0.0479		0.0112	
	yes/yes	0.0168	0.36	0.0078	0.38
	no/yes	0.8943	0.01	0.5161	-0.06
	*no/no*	*	*	*	*
animals sold		0.0531	0.00		

* No p-value and estimate for reference level

** For explanation of factor levels see “Discussion/Impact of farm and management characteristics”

*** Within the scope of the final cleaning of the stables

With regard to the metric variables, it is noticeable that above all, monetary factors such as production costs or costs for veterinary service seem to play an important role. Thus, in the unifactorial approach, increasing production costs per kg live weight gain (LWG) had the effect of poorer scores for MOR and ADG. However, when the influence of other significant factors is taken into account, production costs no longer seem to have such a strong effect on ADG and are even associated with a considerably lower mortality. Poorer performance in MOR is also reflected by higher veterinary costs. An increase in feed intake per pig and day is reflected in significantly better daily gain and better scores on the EXT.

### On-farm health indicators

Contrasting the characteristics under study by age group showed that significant differences in prevalence level occurred for ten of the remaining on-farm indicators (Table 5). Tail biting lesions, ear haematoma and coughing index did not vary substantially between the age groups. Bursa auxiliaris, bursitis, faecal skin dirtying, ophthalmic discharge, conjunctivitis and lameness were more often found in age group 2, whereas skin lesions, purulent nasal discharge, flank biting lesions, ear biting lesions and pigs showing signs of diarrhoea at the pen level showed a higher prevalence in age group 1.

**Table 5:** Mean prevalence [%] at the pen level of on-farm indicators and p-value of the t-test for age group 1 (n = 669 pens) and age group 2 (n = 532 pens)

Indicator	age group 1		age group 2		p-value	correction factor
	**mean**	**SD**	**Mean**	**SD**		
bursa auxiliaris	63.0	23.4	76.4	17.8	**< 0.0001**	1.21
bursitis	1.7	3.7	2.4	4.4	**0.0012**	1.41
faecal skin dirtying	9.0	16.6	12.2	18.6	**0.0020**	1.36
skin lesions	5.5	9.9	3.0	6.6	**< 0.0001**	0.54
purulent nasal discharge	2.2	4.8	1.3	3.3	**0.0002**	0.59
ophthalmic discharge	4.8	7.7	8.9	12.7	**< 0.0001**	1.85
conjunctivitis	2.9	7.4	5.0	10.3	**< 0.0001**	1.72
flank biting lesions	2.2	5.9	0.7	2.9	**< 0.0001**	0.32
tail biting lesions	2.7	8.2	3.2	8.6	0.2694	-*
ear haematoma	1.3	3.5	1.6	3.3	0.1415	-*
ear biting lesions	3.1	7.5	0.9	4.9	**< 0.0001**	0.29
lameness	1.7	3.7	2.3	4.6	**0.0091**	1.35
coughing index	1.3	0.1	1.6	0.1	0.0843	-*

* No correction factor calculated because of nonsignificant t-test

Taking the adjustment of Table 5 into account, it was found that Bursa auxiliaris was by far the most frequently observed finding (76.0 % ± 18.3 %). This was followed by faecal skin dirtying (12.4 % ± 15.7 %) and ophthalmic discharge (8.9 % ± 9.9 %), whereas flank biting lesions were found the least (0.7 %± 1.3 %). In general, it could be stated that most prevalence levels were very low (< 5 %) and therefore showed a right-skewed distribution, which justifies logittransformation and zstandardization. Detailed results are shown in Table 6, and the observed prevalence of all on-farm indicators can be found in Additional file 1.

**Table 6:** Descriptive measures of selected health indicators from on-farm health information in the study collective of 154 pig units (farm-specific prevalence, corrected by age group)

Indicator	unit	mean	median	SD	CV	min	max
bursa	%	76.0	80.8	18.3	24.1	10.5	100.0
bursitis	%	2.4	1.9	2.2	90.6	0.0	13.2
faecal skin dirtying	%	12.4	5.6	15.7	126.7	0.0	89.0
skin lesions	%	2.8	1.6	3.6	126.5	0.0	21.5
purulent nasal discharge	%	1.3	0.9	1.6	124.8	0.0	10.0
ophthalmic discharge	%	8.9	5.9	9.9	110.6	0.0	59.3
conjunctivitis	%	4.9	1.1	8.6	176.7	0.0	45.0
flank biting lesions	%	0.7	0.0	1.3	176.2	0.0	8.1
tail biting lesions*	%	2.9	1.6	4.2	147.1	0.0	25.7
ear haematoma*	%	1.5	1.1	1.5	105.6	0.0	8.4
ear biting lesions	%	0.9	0.3	1.5	181.2	0.0	12.1
lameness	%	2.3	1.6	2.2	98.3	0.0	10.6
coughing index*^,^**	bouts/min	0.05	0.03	0.06	118.0	0.0	0.4
diarrhoea	%	8.3	0.0	16.3	195.7	0.0	87.5

* Observed prevalence (no correction factor because of nonsignificant t-test)

** Number of coughing bouts in 2 × 3 min, divided by number of animals per pen

**Fig. 1:**
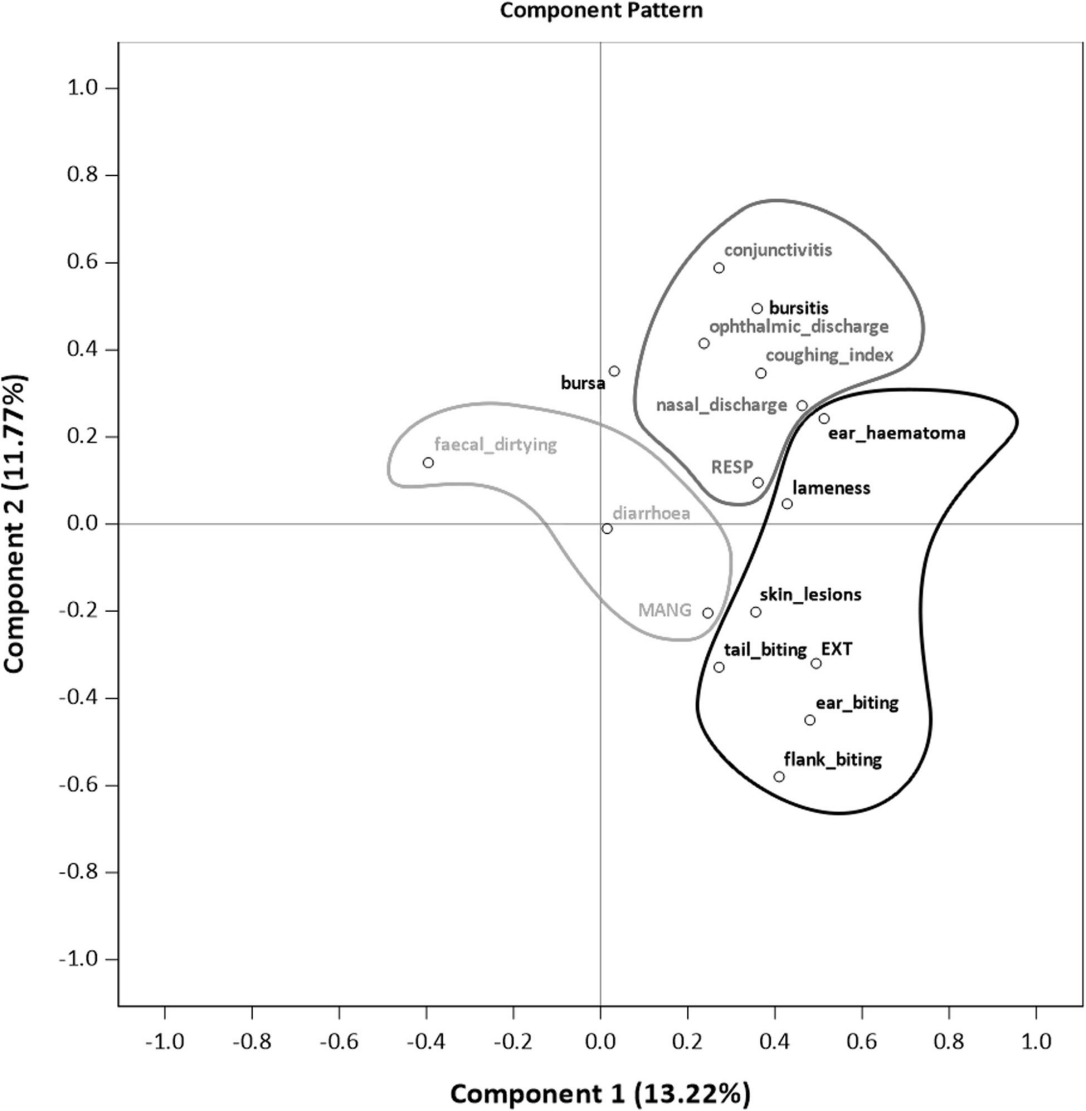
Principal component analysis plot of selected on-farm indicators and health scores based on findings in slaughtered pigs (*RESP* respiratory lesions, grey; *EXT* exterior lesions, black; *MANG* animal management, light grey

The majority of the Spearman correlation coefficients for on-farm and slaughter indicators lay beneath 0.2 and the highest coefficient was calculated for tail biting lesions recorded on-farm and at slaughter with 0.33. Nevertheless, the factor map of the principal component analysis of selected on-farm indicators and slaughter scores (Fig. 1) underlines the technical association between scores based on findings in slaughtered pigs and corresponding indicators in living animals. However, Bursa auxiliaris and bursitis were spotted at some distance from the other exterior lesions, and RESP was found to be closer to lameness than to the respiratory indicators. The first component explains 13.2 %, and the second component explains 11.8 % of the total variance.

The results of the unifactorial ANOVA revealed that none of the selected on-farm indicators could predict any slaughter score to a higher degree than 8.26 % (R² of model “EXT = ear haematoma”), and the majority of R² values were lower than 1 % (Table 7). Ear haematoma and faecal skin dirtying showed significant p-values in two models, whereas skin lesions, flank biting lesions, tail biting lesions, ear biting lesions and lameness each predicted one score significantly, and the remaining factors showed no significant p-value. Therefore, these factors may be considered potential supplemental indicators for health monitoring, as scores based on findings in slaughtered pigs cannot be depicted by any of them.

**Table 7:** Results of unifactorial analyses for on-farm indicators and selected health scores (age group corrected, logit- and z-transformed, farm specific prevalence). Significant p-values and corresponding indicators are bold

indicator	RESP		EXT		MANG	
	**p-value**	**R²**	**p-value**	**R²**	**p-value**	**R²**
bursa auxiliaris	0.4871	0.32 %	0.7251	0.08 %	0.5778	0.20 %
bursitis	0.9683	0.00 %	0.2307	0.94 %	0.5673	0.22 %
**faecal skin dirtying**	0.4393	0.39 %	**0.0155**	3.80 %	**0.0168**	3.70 %
**skin lesions**	0.4036	0.46 %	**0.0066**	4.75 %	0.0587	2.33 %
purulent nasal discharge	0.1004	1.77 %	0.4304	0.41 %	0.3835	0.50 %
ophthalmic discharge	0.7804	0.05 %	0.7521	0.07 %	0.3270	0.63 %
conjunctivitis	0.2180	1.00 %	0.2741	0.79 %	0.5917	0.19 %
**flank biting lesions**	0.1635	1.27 %	**0.0071**	4.67 %	0.7537	0.06 %
**tail biting lesions**	0.8921	0.01 %	**0.0121**	4.07 %	0.2554	0.85 %
**ear haematoma**	**0.0306**	3.04 %	**0.0003**	8.26 %	0.2598	0.83 %
**ear biting lesions**	0.0987	1.78 %	**0.0011**	6.82 %	0.3282	0.63 %
**lameness**	0.4515	0.37 %	**0.0337**	2.93 %	0.1237	1.55 %
coughing index	0.0712	2.13 %	0.4843	0.32 %	0.5382	0.25 %
diarrhoea	0.4208	0.43 %	0.3284	0.63 %	0.5431	0.24 %

EXT was the score with the highest number of significant associations with on-farm indicators. There was also a great degree of professional agreement, since all observed indicators concerning exterior lesions, despite bursa and bursitis, predicted EXT significantly. In contrast, RESP indicated a significant association with ear haematoma only, but none of the respiratory indicators and MANG could be significantly predicted by only faecal skin dirtying.

## Discussion

Animal-based indicators may be an important tool for health monitoring, although they cannot replace but supplement good stockmanship or consultancy for early detection of herd health problems [9]. The present study provides insight into the different management and farm characteristics of 154 typical German fattening pig units and their influence on established health scores. Furthermore, an interconnection between selected health scores and welfare indicators recorded on-farm was analysed.

### Herd selection and availability of data

Based on the results of Grosse-Kleimann et al. [2], only those farms were taken into account that started the fattening period, with pigs having an initial body weight of 24 to 33.5 kg. This bodyweight range is typical in Germany and therefore implemented by the majority of fattening pig farmers. As the health scores in the categories above and below this weight range deviate strongly, the results of the study can only be related to this collective and would have to be validated for pigs of other weight or age by means of additional investigations.

The production data were collected in a harmonized way by the VzF advisors as part of the farm sector analysis and were processed for the project especially as described in a previous publication. For the level classification of categorical factors, it must be kept in mind that a level fulfils the situation on a farm to at least 90 %. This means that a certain inaccuracy in the data cannot be ruled out. Since many pig units have grown over a long period, a 100 % accurate classification is often not possible.

To record the status quo of the study herds, on-farm health information was acquired. Since the aim of the study was to develop factors for monitoring that are easy to implement under practical conditions, each pig unit was visited only once, and the fattening pigs from a maximum of eight randomly selected pens were examined. In contrast, the production data and health scores refer to the aggregated data of a complete half-year and take into account all pigs that were kept or sold for slaughter during this period. However, with regard to the whole study collective, the large number of visited farms and pigs statistically compensates for this. Hence, to obtain a rough impression of the health status of individual pig herds, the survey is indicated to be suitable.

### Impact of farm and management characteristics

A positive impact on pig performance through record keeping by advisory services in general was demonstrated by van Staaveren et al. [10]. Pandolfi et al. [11] found that farm and management characteristics, such as the ventilation system or group size, can act as potential starting points for improving animal welfare. For the present study, comparison of uni- and multifactorial analyses indicates an association of several factors with the given health scores. However, in an overall context, regarding the other characteristics under study, confounding changes the risk perspective. Since one aim of the study was to find the set of farm characteristics for each health score that showed the most significant influence and could be usable as regulation screws to improve pig health, the focus was on multifactorial analyses.

Mortality is generally accepted as an indicator in most epidemiological studies describing animal health [8, 12–14]. Here, MOR is primarily associated with direct cost-free benefits as well as production costs: the higher the number of animals that died or were culled pre-slaughter, the lower the profits and the more production costs per kilogram of carcass sold arise. Other farm characteristics, however, did not show a significant influence in the multifactorial analysis in the study collective. In contrast, Agostini et al. [15] found that multiple origins of pigs led to higher mortality. They also observed lower mortality in pigs with higher IBW, which is in line with the present study. An investigation of grow-finishing pigs in Belgium [16] also identified the origin of piglets and furthermore the duration of fattening period and season for the beginning of the fattening period as risk factors for higher mortality. Similar results were shown by Oliveira et al. [17]. The different results reveal that the mortality rate is a multicausal event that is difficult to attribute to one specific reason or farm characteristic.

Although average daily gain is a non-animal-based measure, it could be a sign of animal health problems and therefore is an important indicator for an overall assessment of pig health [18]. The results of the present study showed that the ADG is the score that is by far the most influential or predictable by the production data. Goodness of fit well over 90 % can be achieved through modelling with the feed intake per day, the feed energy per kg of LWG and the production costs. These variables are closely related in terms of content, as production costs largely consist of feed costs and thus the daily feed intake and feed energy, respectively. However, these are exclusively variable and measurable factors and not farm-specific conditions that can be adjusted to influence the target value. Nevertheless, during a consultation, it is possible to determine which of the three factors is not within the normal range and use this information as a starting point for improvement in daily weight gain. Other studies that examined the influence of farm characteristics on average daily gain did not find a significant effect of group size [19] or castration (physical and via vaccination) [20] but a positive impact of straw bedding on average daily gain [21].

The use of slaughter findings as a monitoring and surveillance tool for pig health is frequently implemented in Europe [22–24]. However, the influence of farm characteristics on findings in slaughtered pigs has not been consistently confirmed. Kongsted et al. [25] showed that the production system has an impact on several health-related lesions, whereas Cagienard et al. [26] could not find significant differences in meat inspection data related to the type of housing.

In the current study, regarding the animal-based slaughter score EXT, the herd attributes type of buying in criteria and fattening boars stayed in the final models as significant effects. Type B (German piglets with BHZP as main boar breed and DAN as main sow breed) and E (German piglets with mixed boar and sow breeds) farms seem to have fewer exterior lesions than the reference group. However, it must be taken into account that the comparison groups in this study were relatively small, with 12 and 13 farms, respectively; therefore, the significance of the results needs to be interpreted with caution. The fattening of boars also plays a role in exterior lesions. Farms that housed entire male pigs had more problems with external injuries, such as skin lesions or tail and ear biting lesions. This is in line with common literature, which found increased sexual and aggressive behaviour against pen mates in single-sex male groups [27, 28]. Concerning metric FC, the intake of feed per pig and day seem to have a protective influence on EXT. The suggestion is that satisfied animals are lazier and therefore show less aggression against pen mates.

### Reflection of on-farm information in health scores

One aim of the study was to develop health indicators for monitoring that are easy to implement under practical conditions. Therefore, no repeated survey was carried out on the farms, and only a random sample of individual pigs inspected was investigated. In contrast, the production data and health scores refer to the collected data of a complete half-year and take into account all animals that were kept or sold during this period. This aspect is underlined by the relatively low R² of the unifactorial on-farm health information models (Table 8). Nevertheless, veterinary examination can give an impression of the health status of individual pig herds and provide clues as to which health problems are not represented by routine processes along the pig production chain [29].

The results of official meat inspection are, on the one hand, animal-based, but on the other hand, the lifetime health status is displayed in a way such that special health events are hidden by aggregation.

The idea of comparing the actively recorded health status of living animals with assessments based on slaughter findings has already been pursued several times. Maisano et al. [30] detected similar prevalence levels of animal-based measures on-farm and slaughter plants, whereas Carroll et al. [31] were able to establish little correlation between health problems in live animals and skin and tail lesions that developed at least ten weeks before slaughter. Van Staaveren et al. [32] also identified skin and tail lesions as “potential iceberg indicators” at the slaughterhouse to replace on-farm assessments. This fits with the results from the present study, as skin lesions and bite lesions on ears, tail and flanks were also significantly mapped by EXT, and a small correlation was found for tail biting lesions on farm and tail lesions detected at slaughter.

The respiratory tract findings, on the other hand, showed only a small statistical correlation between indicators at the slaughter plant and the corresponding indicators at the farm. Other studies that investigated the association of lung or pleural lesions and on-farm indicators for respiratory diseases also found no or little significance [33, 34]. Even though the principal component analysis supports a substantial relationship, RESP could be significantly modelled by blood ears only and none of the technically assigned ones, e.g., coughing index or purulent nasal discharge. One possible explanation for this may be that respiratory lesions in slaughter pigs are often caused by enzootic pneumonia (*Mycoplasma hyopneumoniae*) [35], which is typically clinically apparent from the sixth to the eighth fattening week [36]. As the study included pigs from the first to the twelfth fattening week, the typical time period for coughing caused by enzootic pneumonia was underrepresented.

MANG could also scarcely be modelled by the on-farm indicators. However, this is to some extent reasonable, as the score includes, among others, the indicator “dermal damage by handling”, which occurs during transport from the farm to the slaughterhouse and therefore is not expected to be found during on-farm examination. Another score component, liver milk spots, usually remains clinically inconspicuous in fattening pigs [36, 37] and therefore cannot show connection to the on-farm indicators.

In addition to respiratory on-farm indicators (ophthalmic and nasal discharge, conjunctivitis and coughing index), Bursa auxiliaris, bursitis and diarrhoea showed no significant association with any of the slaughter scores. From this, the recommendation can be derived to consider these indicators in addition to the slaughter findings for health monitoring.

## Conclusions

In general, the present study shows that the investigated health scores are influenced to varying degrees by different farm and management characteristics. However, many of the influencing variables examined have a stronger influence when considered individually, but they do not longer play a decisive role in the multifactorial context. In practice, this confounding effect suggests that the whole farm with all its characteristics should always be taken into account for advising and changing of just individual factors has to be avoided.

From a specific perspective, ADG can be explained in a multifactorial model to almost 100 % by production costs and feed intake, whereas models for animal-based scores such as MOR or EXT had a much lesser coefficient of determination. Hence, MOR and EXT seem to be more important for describing animal welfare.

With regard to the on-farm indicators, a general contextual connection could be established via principal component analysis. In connection with the associated slaughter scores, however, it became apparent that only EXT could be mapped significantly by the result of the survey on the farms. Following this, the on-farm collection of respiratory indicators, e.g., coughing index or purulent nasal discharge as well as bursa or bursitis and diarrhoea, could be recommended as supplemental items for health monitoring.

Overall, it can be stated that production data are suitable to map health issues of fattening pigs in the context of monitoring and to obtain an impression of possible adjusting screws for consultancy in the next step. In this study, the production data are based on aggregated data of a sixth month period, and the on-farm examination of pigs may provide additional information in the form of a snapshot.

## Supplementary information

Additional file 1 Descriptive results of all farm characteristics-factors and on-farm indicators.

Additional file 2 Results of uni- and multifactorial analyses for all health scores and farm characteristics.

## Data Availability

The data were collected on an individual basis from farmers and slaughterhouses. Each participant gave written consent with the understanding that data would not be transferred to a third party. Therefore, any data transfer to interested persons is not allowed without an additional formal contract. Data are available to qualified researchers who sign a contract with the University of Veterinary Medicine Hannover and VzF. This contract will include guarantees to the obligation to maintain data confidentiality in accordance with the provisions of the German data protection law. Currently, there exists no data access committee or other body who could be contacted for the data. However, for this purpose, a committee will be founded. This future committee will consist of the authors as well as members nominated by the University of Veterinary Medicine Hannover and the VzF. Interested cooperative partners who are able to sign a contract as described above may contact: Prof. Dr. Lothar Kreienbrock Department of Biometry, Epidemiology and Information Processing University of Veterinary Medicine, Hannover Bünteweg 2, 30559 Hannover Email: lothar.kreienbrock@tiho-hannover.de

## Abbreviations

ADG: average daily gain score
BHZP: German Federal Hybrid Breeding Programme
EXT: exterior lesions score
FCR: feed conversion ratio score
LWG: live weight gain
MANG: animal management score
MOR: mortality score
PIC: Pig Improvement Company
RESP: respiratory lesions score
TF: treatment frequency score
VzF: Association for Promoting Farming Economics
