# Supplementary material for: Health Monitoring of Fattening Pigs – Use of Production Data, Farm Characteristics and On-Farm Examination
Source: Porcine Health Manag. 2021 Aug 3;7:45. doi: 10.1186/s40813-021-00225-y (PMC8330030; doi:10.1186/s40813-021-00225-y)
Supplement: Supplementary file 1 — Descriptive results of all farm characteristics-factors and on-farm indicators. [file 40813_2021_225_MOESM1_ESM.docx]

**Descriptive results of all farm characteristics-factors and on-farm indicators**

**Table 1** Descriptive measures of categorical farm characteristics-factors in the study collective of 154 pig units (*BHZP* German Federal Hybrid Breeding Programme, *PIC* Pig Improvement Company, *AIAO* all-in-all-out)

| **factor** | **levels** | **amount** | **N** |
| --- | --- | --- | --- |
| **type of buying in criteria*** | A | 27.3 % | 42 |
|  | B | 23.4 % | 36 |
|  | C | 7.8 % | 12 |
|  | D | 12.3 % | 19 |
|  | E | 13.0 % | 20 |
|  | F | 8.4 % | 13 |
|  | G | 7.8 % | 12 |
| **boar fattening/single sex groups** | no/no | 48.1 % | 74 |
|  | no/yes | 36.4 % | 56 |
|  | yes/yes | 11.7 % | 18 |
|  | (yes/no)** | (3.9 %)* | (6)* |
| **purchase management/pig flow**  (farm(s) of origin/AIAO) | 1 farm/compartmentwise | 59.1 % | 91 |
|  | 1 farm/stablewise | 27.3 % | 42 |
|  | > 1 farm/compartmentwise | 7.8 % | 12 |
|  | (> 1 farm/stablewise)** | (5.8 %)* | (9)* |
| **floor type** | partly slatted | 6.5 % | 10 |
|  | fully slatted | 81.8 % | 126 |
|  | misc. | 11.7 % | 18 |
| **space per FP** | 0.75 m^2^ | 55.8 % | 86 |
|  | 0.825 m^2^ | 32.5 % | 50 |
|  | > 0.9 m^2^ | 10.4 % | 16 |
|  | (misc.)** | (1.3 %)* | (2)* |
| **group size** | 1 - 12 pigs | 30.5 % | 47 |
|  | 13 - 20 pigs | 31.2 % | 48 |
|  | 21 - 50 pigs | 14.3 % | 22 |
|  | misc. | 24.0 % | 37 |
| **post fattening** | no | 25.3 % | 86 |
|  | yes | 74.0 % | 50 |
|  | (misc.)** | (0.65 %)* | (1) |
| **feed** | cereals | 59.7 % | 92 |
|  | cereals & whey | 18.2 % | 28 |
|  | cereals & misc. | 7.8 % | 12 |
|  | misc. | 14.3 % | 22 |
| **phase feeding** | biphase | 9.1 % | 14 |
|  | triphase | 70.1 % | 108 |
|  | multiphase | 19.5 % | 30 |
|  | (misc.)** | (1.3 %)* | (2)* |
| **feeding techniques** | mash | 40.9 % | 70 |
|  | liquid | 45.5 % | 63 |
|  | misc. | 10.4 % | 16 |
|  | (dry)** | (3.3 %)* | (5)* |
| **feed availability** | rationed | 54.6 % | 84 |
|  | ad libitum | 36.4 % | 56 |
|  | misc. | 9.1 % | 14 |
| **phosphor reduction** | yes | 27.3 % | 42 |
|  | no | 67.5 % | 104 |
|  | (misc.)** | (5.2 %)* | (8)* |
| **feed energy** | < 13 MJ | 39.0 % | 60 |
|  | 13 - 13.4 MJ | 59.7 % | 92 |
|  | (misc.)** | (1.3 %)* | (2)* |
| **(drinker type)** | (cup drinker)** | (3.9 %)* | (6)* |
|  | nipple drinker | 93.5 % | 144 |
|  | (misc.)** | (2.6 %)* | (4)* |
| **drinking water supply** | private well | 58.4 % | 90 |
|  | private well, treated water | 7.1 % | 11 |
|  | public | 33.8 % | 52 |
|  | (misc.)** | (0.7 %)* | (1)* |
| **climate check** | yes | 27.3 % | 42 |
|  | no | 72.7 % | 112 |
| **addition of acids** | yes | 55.2 % | 85 |
|  | no | 44.8 % | 69 |

* For explanation of factor levels see "Discussion/Impact of farm and management characteristics"

** Factor levels with less than ten pig units were omitted for analyses to avoid extended variation due to sparse data

**Table 2** Descriptive measures of metric farm characteristics-factors in the study collective of 154 pig units (*BFCD* benefits free of direct costs, *LWG* live weight gain)

| **factor** | **unit** | **mean** | **median** | **SD** | **CV** | **min** | **max** |
| --- | --- | --- | --- | --- | --- | --- | --- |
| fattening pig places (FPs) | # | 1,153 | 1,100 | 582 | 51 | 160 | 3,360 |
| animals sold | # | 1,539 | 1,385 | 901 | 59 | 91 | 4,961 |
| pigs/FP | # | 1.31 | 1.29 | 0.31 | 24.13 | 0.53 | 2.15 |
| live weight losses/pig | kg | 66.86 | 65.15 | 9.69 | 14.50 | 45.20 | 97.20 |
| weight gain/pig | kg | 93.49 | 93.79 | 3.50 | 3.75 | 84.40 | 104.27 |
| live weight at sale/pig | kg | 122.67 | 122.66 | 2.93 | 2.39 | 113.17 | 131.31 |
| slaughter weight/pig | kg | 96.72 | 96.80 | 2.18 | 2.25 | 91.12 | 103.73 |
| production costs/kg LWG | € | 0.69 | 0.69 | 0.06 | 9.32 | 0.57 | 0.91 |
| BFDC/100 kg LWG^1^ | € | 18.14 | 19.10 | 9.43 | 51.97 | -13.27 | 38.00 |
| costs for veterinary service/pig | € | 1.00 | 0.60 | 1.17 | 116.27 | 0.02 | 8.33 |
| costs for disinfection*/pig | € | 0.27 | 0.23 | 0.23 | 86.98 | - | 1.22 |
| costs for energy/pig | € | 3.05 | 2.55 | 1.58 | 51.84 | 0.84 | 8.44 |
| feed consumption/pig/day | kg | 2.40 | 2.39 | 0.21 | 8.63 | 1.90 | 3.10 |
| feed energy/kg LWG^1^ | MJ | 37.40 | 37.39 | 2.59 | 6.92 | 31.71 | 44.20 |

* Within the scope of the final cleaning of the stables

**Table 3** Descriptive measures of health indicators from OF in the study collective of 154 pig units (observed, farm specific prevalence)

| indicator | unit | mean | median | SD | CV | min | max |
| --- | --- | --- | --- | --- | --- | --- | --- |
| pale animals | % | 0.2 | - | 0.6 | 336.4 | - | 7.6 |
| runts | % | 0.3 | - | 0.6 | 226.7 | - | 3.9 |
| bursa auxiliaris | % | 69.0 | 71.9 | 17.1 | 24.8 | 8.7 | 95.3 |
| bursitis | % | 2.0 | 1.6 | 1.8 | 88.3 | - | 9.7 |
| faecal skin dirtying | % | 10.6 | 4.8 | 13.5 | 127.1 | - | 66.6 |
| skin lesions | % | 4.5 | 2.6 | 5.4 | 120.5 | - | 30.2 |
| purulent nasal discharge | % | 1.8 | 1.1 | 2.4 | 131.6 | - | 15.6 |
| ophthalmic discharge | % | 6.6 | 4.4 | 7.7 | 116.2 | - | 55.7 |
| conjunctivitis | % | 3.7 | 1.0 | 6.6 | 175.5 | - | 42.4 |
| abscess | % | 0.6 | 0.1 | 0.9 | 159.1 | - | 6.8 |
| hernia | % | 1.0 | 0.7 | 1.3 | 127.2 | - | 7.4 |
| flank biting lesions | % | 1.6 | - | 3.0 | 183.0 | - | 16.7 |
| abnormal breathing | % | 0.2 | - | 0.5 | 238.8 | - | 3.2 |
| tail biting lesions | % | 2.9 | 1.6 | 4.2 | 147.1 | - | 25.7 |
| loss of substance at tail | % | 1.4 | - | 3.1 | 227.7 | - | 25.9 |
| ear haematoma | % | 1.5 | 1.1 | 1.5 | 105.6 | - | 8.4 |
| ear biting lesions | % | 2.1 | 1.0 | 3.2 | 150.3 | - | 18.8 |
| loss of substance at ears | % | 8.0 | 5.6 | 8.4 | 104.5 | - | 52.3 |
| ear necrosis | % | 31.0 | 27.8 | 15.7 | 50.5 | 3.1 | 82.6 |
| lameness | % | 1.9 | 1.3 | 1.9 | 98.6 | - | 9.1 |
| coughing index* | bouts/min | 0.05 | 0.03 | 0.06 | 118.0 | 0.0 | 0.4 |
| diarrhoea | % | 8.3 | 0.0 | 16.3 | 195.7 | 0.0 | 87.5 |

* Number of coughing bouts in 2 x 3 minutes. divided by number of animals per pen
