# Supplementary material for: Health Monitoring of Fattening Pigs – Use of Production Data, Farm Characteristics and On-Farm Examination
Source: Porcine Health Manag. 2021 Aug 3;7:45. doi: 10.1186/s40813-021-00225-y (PMC8330030; doi:10.1186/s40813-021-00225-y)
Supplement: Supplementary file 2 — Results of uni- and multifactorial analyses for all health scores and farm characteristics. [file 40813_2021_225_MOESM2_ESM.docx]

**Results of uni- and multifactorial analyses for all health scores and farm characteristics**

**Table 1** Results of unifactorial analyses (factors with p‑value < 0.1) and final multifactorial model (factors with p‑value < 0.05) with R² for all health scores. Factors remaining in final model are in bold letters, and the reference level is in italic letters (*BFDC* benefits free of direct costs, *AIAO* all-in-all-out, *LWG* live weight gain, *FP* finishing pig place, *BHZP* German Federal Hybrid Breeding Programme, *PIC* Pig Improvement Company).

| **score** | **R²** | **factor** | **factor levels** | **unifactorial** | | **multifactorial** | |
| --- | --- | --- | --- | --- | --- | --- | --- |
|  |  |  |  | **p-value** | **estimate** | **p-value** | **estimate** |
| **MOR** | **17.54 %** | **BFDC/100 kg LWG** |  | <.0001 | -0.04 | <.0001 | -0.05 |
|  |  | **feed energy/kg LWG** |  | 0.0161 | 0.08 | 0.0314 | 0.08 |
|  |  | feed availability |  | 0.0570 |  |  |  |
|  |  |  | misc. | 0.7548 | 0.09 |  |  |
|  |  |  | ad libitum | 0.0175 | 0.42 |  |  |
|  |  |  | *rationed* | * | * |  |  |
|  |  | **costs for veterinary service/pig** |  | 0.0672 | 0.13 | 0.0482 | 0.14 |
|  |  | **production costs/kg LWG** |  | 0.0813 | 2.23 | 0.0043 | -5.60 |
|  |  | feed energy |  | 0.0992 |  |  |  |
|  |  |  | < 13 MJ | 0.0992 | 0.28 |  |  |
|  |  |  | *13 - 13.4 MJ* | * | * |  |  |
| **ADG** | **96.10 %** | **feed consumption/pig/day** |  | <.0001 | -3.49 | <.0001 | -4.31 |
|  |  | **feed energy/kg LWG** |  | <.0001 | 0.18 | <.0001 | 0.24 |
|  |  | group size |  | <.0001 |  |  |  |
|  |  |  | misc. | 0.0227 | 0.46 |  |  |
|  |  |  | 21 - 50 pigs | 0.0144 | -0.58 |  |  |
|  |  |  | 13 - 20 pigs | 0.0034 | -0.56 |  |  |
|  |  |  | *1 - 12 pigs* | * | * |  |  |
|  |  | space per FP |  | <.0001 |  |  |  |
|  |  |  | > 0.9 m^2^ | <.0001 | -1.14 |  |  |
|  |  |  | 0.825 m² | 0.0277 | -0.37 |  |  |
|  |  |  | *0.75 m^2^* | * | * |  |  |
|  |  | type of buying in criteria** |  | 0.0001 |  |  |  |
|  |  |  | G | 0.0142 | 0.75 |  |  |
|  |  |  | F | 0.0010 | -0.99 |  |  |
|  |  |  | E | 0.0507 | -0.50 |  |  |
|  |  |  | D | 0.2479 | 0.30 |  |  |
|  |  |  | C | 0.8341 | -0.06 |  |  |
|  |  |  | B | 0.5755 | -0.12 |  |  |
|  |  |  | *A* | * | * |  |  |
|  |  | pigs/FP |  | 0.0011 | -0.83 |  |  |
|  |  | feeding techniques |  | 0.0022 |  |  |  |
|  |  |  | misc. | 0.1604 | 0.38 |  |  |
|  |  |  | mash | 0.0073 | -0.46 |  |  |
|  |  |  | *liquid* | * | * |  |  |
|  |  | water supply |  | 0.0049 |  |  |  |
|  |  |  | public | 0.0012 | -0.56 |  |  |
|  |  |  | private well, treated water | 0.6106 | -0.16 |  |  |
|  |  |  | *private well* | * | * |  |  |
|  |  | **production costs/kg LWG** |  | 0.0083 | 3.27 | <.0001 | 1.29 |
|  |  | floor type |  | 0.0145 |  |  |  |
|  |  |  | misc. | 0.0048 | 0.70 |  |  |
|  |  |  | partially slatted | 0.3237 | 0.32 |  |  |
|  |  |  | *fully slatted* | * | * |  |  |
|  |  | feed availability |  | 0.0192 |  |  |  |
|  |  |  | misc. | 0.3581 | 0.26 |  |  |
|  |  |  | ad libitum | 0.0177 | -0.40 |  |  |
|  |  |  | *rationed* | * | * |  |  |
|  |  | live weight losses/pig |  | 0.0227 | -0.02 |  |  |
|  |  | boar fattening single sex |  | 0.0245 |  |  |  |
|  |  |  | yes/yes | 0.8974 | 0.03 |  |  |
|  |  |  | no/yes | 0.0103 | -0.44 |  |  |
|  |  |  | *no/no* | * | * |  |  |
|  |  | slaughter weight/pig |  | 0.0272 | -0.08 |  |  |
|  |  | BFDC/100 kg LWG |  | 0.0311 | -0.02 |  |  |
|  |  | post fattening |  | 0.0478 |  |  |  |
|  |  |  | yes | 0.0478 | 0.37 |  |  |
|  |  |  | *no* | * | * |  |  |
|  |  | weight gain/pig |  | 0.0890 | -0.04 |  |  |
| **FCR** | **93.22 %** | **feed energy/kg LWG** |  | < 0.0001 | 0.37 | < 0.0001 | 0.33 |
|  |  | **production costs/kg LWG** |  | < 0.0001 | 1.02 | < 0.0001 | 1.99 |
|  |  | BFDC/100 kg LWG |  | < 0.0001 | -0.05 |  |  |
|  |  | **feed consumption/pig/day** |  | 0.0004 | 1.36 | 0.0434 | 0.22 |
|  |  | floor type |  | 0.0524 |  |  |  |
|  |  |  | misc. | 0.0156 | 0.60 |  |  |
|  |  |  | partially slatted | 0.6903 | 0.13 |  |  |
|  |  |  | *fully slatted* | * | * |  |  |
|  |  | feed energy |  | 0.0734 |  |  |  |
|  |  |  | < 13 MJ | 0.0734 | 0.29 |  |  |
|  |  |  | *13 - 13.4 MJ* | * | * |  |  |
|  |  | weight gain/pig |  | 0.0790 | -0.04 |  |  |
|  |  | slaughter weight/pig |  | 0.0792 | -0.06 |  |  |
|  |  | group size |  | 0.0865 |  |  |  |
|  |  |  | misc. | 0.0333 | 0.46 |  |  |
|  |  |  | 21 - 50 pigs | 0.8133 | 0.06 |  |  |
|  |  |  | 13 - 20 pigs | 0.8070 | -0.05 |  |  |
|  |  |  | *1 - 12 pigs* | * | * |  |  |
| **TF** | **18.75 %** | **costs for veterinary service/pig** |  | < 0.0001 | 0.33 | < 0.0001 | 0.31 |
|  |  | **feeding techniques** |  | 0.0073 |  | 0.0346 |  |
|  |  |  | mash | 0.0028 | 0.83 | 0.0102 | 0.67 |
|  |  |  | liquid | 0.9754 | -0.01 | 0.6736 | 0.07 |
|  |  |  | *misc.* | * | * | * | * |
|  |  | costs for disinfection***/pig |  | 0.0113 | 0.88 |  |  |
|  |  | addition of acids |  | 0.0316 |  |  |  |
|  |  |  | yes | 0.0316 | 0.35 |  |  |
|  |  |  | *no* | * | * |  |  |
|  |  | feed consumption/pig/day |  | 0.0432 | -0.80 |  |  |
|  |  | weight gain/pig |  | 0.0545 | 0.04 |  |  |
|  |  | feed availability |  | 0.0773 |  |  |  |
|  |  |  | rationed | 0.8161 | 0.07 |  |  |
|  |  |  | ad libitum | 0.0333 | -0.37 |  |  |
|  |  |  | *misc.* | * | * |  |  |
| **RESP** | **20.32 %** | **type of buying in criteria**** |  | 0.0001 |  | < 0.0001 |  |
|  |  |  | G | 0.0090 | 0.66 | 0.0076 | 0.66 |
|  |  |  | F | 0.0005 | 0.86 | 0.0002 | 0.93 |
|  |  |  | E | 0.1494 | 0.30 | 0.0849 | 0.36 |
|  |  |  | D | 0.0196 | 0.50 | 0.0064 | 0.58 |
|  |  |  | C | 0.0482 | -0.50 | 0.1795 | -0.34 |
|  |  |  | B | 0.0620 | 0.32 | 0.0217 | 0.41 |
|  |  |  | *A* | * | * | * | * |
|  |  | drinking water supply |  | 0.0054 |  |  |  |
|  |  |  | public | 0.0078 | -0.37 |  |  |
|  |  |  | private well, treated water | 0.0183 | -0.61 |  |  |
|  |  |  | *private well* | * | * |  |  |
|  |  | **costs for energy/pig** |  | 0.0200 | 0.10 | 0.0126 | 0.10 |
|  |  | **costs for disinfection***/pig** |  | 0.0597 | -0.53 | 0.0041 | 0.12 |
|  |  | costs for veterinary service/pig |  | 0.0802 | 0.10 |  |  |
| **EXT** | **26.21 %** | **type of buying in criteria**** |  | 0.0014 |  | 0.0024 |  |
|  |  |  | G | 0.6629 | 0.08 | 0.8411 | 0.04 |
|  |  |  | F | 0.0011 | -0.57 | 0.0107 | -0.47 |
|  |  |  | E | 0.9358 | 0.01 | 0.6050 | 0.08 |
|  |  |  | D | 0.4364 | 0.12 | 0.8524 | 0.03 |
|  |  |  | C | 0.0053 | -0.50 | 0.0010 | -0.60 |
|  |  |  | B | 0.5943 | -0.07 | 0.2323 | -0.15 |
|  |  |  | *A* | * | * | * | * |
|  |  | **feed consumption/pig/day** |  | 0.0040 | -0.64 | 0.0400 | -0.48 |
|  |  | phosphor reduction |  | 0.0135 |  |  |  |
|  |  |  | yes | 0.0135 | -0.26 |  |  |
|  |  |  | *no* | * | * |  |  |
|  |  | **costs for disinfection/pig** |  | 0.0137 | -0.49 | 0.0069 | -0.53 |
|  |  | **boar fattening single sex** |  | 0.0479 |  | 0.0112 |  |
|  |  |  | yes/yes | 0.0168 | 0.36 | 0.0078 | 0.38 |
|  |  |  | no/yes | 0.8943 | 0.01 | 0.5161 | -0.06 |
|  |  |  | *no/no* | * | * | * | * |
|  |  | animals sold |  | 0.0531 | 0.00 |  |  |
| **MANG** | **13.31 %** | **costs for disinfection***/pig** |  | 0.0157 | -0.56 | 0.0347 | -0.49 |
|  |  | **boar fattening single sex** |  | 0.0183 |  | 0.0369 |  |
|  |  |  | yes/yes | 0.0050 | 0.50 | 0.0105 | 0.45 |
|  |  |  | no/yes | 0.2467 | 0.14 | 0.5526 | 0.07 |
|  |  |  | *no/no* | * | * | * | * |
|  |  | animals sold |  | 0.0252 | 0.00 |  |  |
|  |  | **space per FP** |  | 0.0316 |  | 0.0194 |  |
|  |  |  | 0.75 m^2^ | 0.0093 | 0.48 | 0.0058 | 0.54 |
|  |  |  | 0.825 m^2^ | 0.7647 | 0.04 | 0.8913 | 0.02 |
|  |  |  | *> 0.9 m^2^* | * | * | * | * |
| **TOTAL** | **44.75 %** | **feed energy/kg LWG** |  | < 0.0001 | 0.08 | < 0.0001 | 0.07 |
|  |  | **BFDC/100 kg LWG** |  | 0.0005 | -0.01 | 0.0180 | -0.01 |
|  |  | production costs/kg LWG |  | 0.0007 | 1.94 |  |  |
|  |  | **costs for veterinary service/pig** |  | 0.0025 | 0.10 | 0.0254 | 0.06 |
|  |  | **feed consumption/pig/day** |  | 0.0063 | -0.50 | < 0.0001 | -0.83 |
|  |  | **floor type** |  | 0.0254 |  | 0.0365 |  |
|  |  |  | misc. | 0.0068 | 0.32 | 0.1153 | 0.15 |
|  |  |  | partially slatted | 0.7793 | 0.04 | 0.0547 | -0.27 |
|  |  |  | *fully slatted* | * | * | * | * |
|  |  | group size |  | 0.0470 |  |  |  |
|  |  |  | misc. | 0.0112 | 0.26 |  |  |
|  |  |  | 21 - 50 pigs | 0.9299 | -0.01 |  |  |
|  |  |  | 13 - 20 pigs | 0.6152 | 0.05 |  |  |
|  |  |  | *1 - 12 pigs* | * | * |  |  |
|  |  | purchase/occupancy management |  | 0.0719 |  |  |  |
|  |  | (farm(s) of origin/AIAO) | 1 farm/compartmentwise | 0.0445 | 0.28 |  |  |
|  |  |  | 1 farm/stablewise | 0.4786 | -0.06 |  |  |
|  |  |  | *> 1 farm/compartmentwise* | * | * |  |  |
|  |  | **type of buying in criteria**** |  | 0.0727 |  | 0.0047 |  |
|  |  |  | G | 0.2908 | 0.16 | 0.2469 | 0.15 |
|  |  |  | F | 0.2575 | -0.17 | 0.5687 | 0.07 |
|  |  |  | E | 0.6217 | 0.06 | 0.3368 | 0.11 |
|  |  |  | D | 0.6479 | 0.06 | 0.8812 | -0.02 |
|  |  |  | C | 0.0146 | 0.37 | 0.0005 | -0.44 |
|  |  |  | B | 0.7253 | 0.04 | 0.6818 | 0.03 |
|  |  |  | *A* | * | * | * | * |

* No p-value and estimate for reference level

** For explanation of factor levels see "Discussion/Impact of farm and management characteristics"

*** Within the scope of the final cleaning of the stables
